# Oral clefts with associated anomalies: findings in the Hungarian Congenital Abnormality Registry

**DOI:** 10.1186/1472-6831-5-4

**Published:** 2005-06-28

**Authors:** Andrea Sárközi, Diego F Wyszynski, Andrew E Czeizel

**Affiliations:** 1 Foundation for the Community Control of Hereditary Diseases, Torokvesz lejto 32, 1026 Budapest, Hungary; 2 Departments of Medicine and of Epidemiology, Boston University School of Medicine, 715 Albany Street, L-320, Boston, MA 02118, USA

## Abstract

**Background:**

Over the years, great efforts have been made to record the frequency of orofacial clefts in different populations. However, very few studies were able to account for the etiological and phenotypic heterogeneity of these conditions. Thus, data of cases with syndromic orofacial clefts from large population-based studies are infrequent.

**Methods:**

Clinically recognized and notified syndromes and associations including cleft lip with or without cleft palate and other congenital anomalies were selected from the Hungarian Congenital Abnormality Registry (HCAR) between 1973 and 1982 and prevalence rates were calculated.

**Results:**

Of 3,110 cases reported as having orofacial clefts, 653 had multiple congenital abnormalities. Of these, 60 (9.2%) had a known etiology (monogenic: 25 or 3.8%, chromosomal: 31 or 4.7%, teratogenic: 4 or 0.6%). Seventy-three subjects (11.2%) had schisis in addition to the oral cleft. Skeletal anomalies were the most common malformations among cases with cleft lip with/without cleft palate (CL/P) and cleft palate (CP). Disorders of the central nervous system and cardiovascular malformations were also frequently associated.

**Conclusion:**

Surveillance systems, such as the HCAR, provide useful information about prevalence rates of congenital anomalies in a population. However, in a field where new syndromes are being discovered and classifications regularly updated, these rates should only be accepted as provisional.

## Background

It has been known for more than 80 years that cleft lip with or without cleft palate (CL/P) and isolated cleft palate (CP), collectively termed oral clefts (OCs), are frequently associated with congenital anomalies [1]. The prevalence of associated anomalies in subjects with OCs varies widely, ranging from 6% to 63%; however, when broken down by subtype, it is clear that they are much more frequent in patients with isolated CP (13–50%) than in patients with cleft lip (CL) (7%-13%) or patients with cleft lip and palate (CLP) (2%-26%) [1-7]. The sources of variation have been recently described by Wyszynski et al. [1] as 1) differences in case definition and inclusion/exclusion criteria, 2) how long after birth cases are examined, 3) variability of clinical expression of associated anomalies, 4) knowledge and technology available to produce syndrome delineation, 5) selection of patients, sources of ascertainment, and sample size, 6) true population differences and changes in frequency over time.

The evaluation of patients with multiple congenital anomalies (MCAs) is of critical importance because 1. all unbalanced autosomal chromosomal aberrations and most gene mutations and teratogens produce syndromes. Therefore, MCAs are sensitive indicators of germinal mutagens and teratogens [8]. 2. The delineation of an MCA-entity facilitates a better understanding of the phenotypic spectrum, prognosis, and origin of the condition. The latter may be of great importance in genetic counseling or to detect new teratogenic agents. 3. Gene mapping efforts for some of these conditions might become feasible after the identification of informative families.

The objective of this paper is to describe the cases with OCs and associated anomalies identified in a large population-based birth defects registry in Hungary.

## Methods

Eligible cases were newborns with OCs and at least one other congenital anomaly identified from the records of the nation-based "Hungarian Congenital Abnormality Registry" (HCAR; [9]) and born between 1973 and 1982. No data were collected after 1982. Notification by physicians of cases with structural birth defects (i.e., congenital abnormalities) to the HCAR was mandatory during that time period. Most cases were notified by obstetricians, since in Hungary virtually all deliveries occur in inpatient obstetric clinics, or from pediatricians who were working in the neonatal units of inpatient obstetric clinics and various inpatient and outpatient pediatric clinics. During the study period, autopsy was required for all infant deaths, and was often practiced for stillborn fetuses. Pathologists sent a copy of each autopsy report to the HCAR if any birth defects were identified. The recorded total (birth + fetal) prevalence of cases with CL/P and with isolated CP was 1.01 and 0.35 per 1,000 newborns (liveborn infants, stillborn and malformed fetuses from electively-terminated pregnancies), respectively. About 95% of cases with CL/P and close to 90% of cases with isolated CP were reported to the HCAR during the 10 years of the study period [9]. Based on the available clinical notations, each case was assigned to one of three categories:

1) Unspecified multiple congenital anomalies. These cases had such limited information that it was not possible to differentiate the type of malformations present. New or supplementary information was requested from the clinicians. Of 6,641 total cases with multiple congenital anomalies reported to the HCAR, 131 subjects (2.0%) were in this category.

2) Unidentified (but specified) multiple congenital anomalies. These cases had information about the associated anomalies, but it was not possible to distinguish between syndrome, sequence, and association. Several attempts were made to remediate this situation: (a) a copy of the detailed necropsy records was requested from the pathologist when the case expired (stillborn or infant death). Pathology records were received in 88% of these cases; (b) cases that listed congenital dislocation of the hip (mainly hip dysplasia or Ortolani positivity), club-foot, congenital inguinal hernia, or some other mild congenital anomaly were also part of a study on postural congenital anomalies [10], which provided additional information; (c) other cases with multiple congenital anomalies who survived the infant period were officially referred to one of the eight Multiple Congenital Anomalies Examination Centers, which functioned as part of the HCAR. Each center had a circumscribed catchment area and was equipped with laboratory facilities suitable to carry out chromosome analysis, serological examinations (ie, for rubella and cytomegalovirus, toxoplasmosis, etc.), and certain biochemical tests. Clinical examination by a dysmorphologist was combined with multidisciplinary counseling concerning possible treatment and prognosis for the patient, as well as regarding the risk of recurrence in further pregnancies [11]; (d) occasionally, the records had sufficient information, but no diagnosis. Of 17 cases with OCs, four cases were eventually identified as having a syndrome (Ectrodactyly-Ectodermal Dysplasia-CL/P, Meckel, Mohr, and Roberts) and one had an association (schisis) [8]. Finally, (e) cases born between 1980 and 1982 were studied further in the context of a separate epidemiological study carried-out between 1982 and 1983. A structured questionnaire and an explanatory letter were mailed to the parents of these cases soliciting information on their children's condition. The information gathered made it possible to confirm or modify some diagnoses. Of 6,641 total cases with multiple congenital anomalies reported to the HCAR, 3,393 subjects (51%) had unidentified but specified MCAs.

3) Identified syndromes or associations. These cases were accepted without any further follow-up on the basis of the clinical records. Of 6,641 total cases with multiple congenital anomalies reported to the HCAR, 3,117 subjects (47%) were in this category.

## Results

The dataset comprised 65,923 cases with congenital anomalies born between 1973 and 1982. The number of livebirths during the study period was 1,667,166, resulting in a prevalence of cases with congenital anomalies of 39.5 per 1,000 or ≈ 4% livebirths. The number of confirmed cases with more than one congenital anomaly was 6,641 (prevalence at birth: 3.98 per 1,000 or 10% of all anomalies). The stillbirth and infant death rates for cases with multiple congenital anomalies were 8.67% and 23.8%, respectively, which are about 10 times higher than the corresponding national figures for the study period.

Of the 6,641 cases with more than one anomaly, 2,341 had a syndrome, 776 an association, 131 were unspecified, and 3,393 were unidentified. Of the 3,110 cases with OCs, 2,457 or ≈ 80% were isolated OCs (nonsyndromic and sequences) and 653 had multiple congenital anomalies (syndromic and associations) (Table 1). In the latter group, 60 (9.2%) had a known etiology (monogenic: 25 or 3.8%, chromosomal: 31 or 4.7%, teratogenic: 4 or 0.6%) (Table 2). There were 73 cases (11.2%) with OCs associated to schisis. The remaining 520 cases with OCs and other anomalies (351 CL/P and 169 CP) were unidentified (*verba*, of unknown etiology).

**Table 1:** Cases with isolated oral clefts (OCs) and with OCs plus other congenital anomalies in the Hungarian Congenital Abnormality Registry (HCAR), 1973–1982.

**Category Group**	**Number**	Prevalence*
*Isolated*		
Cleft lip with or without cleft palate (CL/P)	1,687	1.02
Cleft lip only	607	0.36
Cleft lip and palate	1,080	0.65
Posterior cleft palate only	632	0.38
Robin sequence	99	0.06
ADAM sequence (*n *= 31)		
including atypical oral clefts	10	0.01
Holoprosencephaly (*n *= 38)		
including orofacial clefts	12	0.01
Others (median, oblique, etc.)	17	0.01

**Subtotal**	**2,457**	**1.47**

*Multiple Congenital Anomalies*		
CL/P in recognized entities	83	0.05
CL/P in unidentified entities	351	0.21
CP in recognized entities	48	0.03
CP in unidentified entities	169	0.10
Robin sequence in recognized syndrome	2	0.00

**Total**	**653**	**0.39**

**All cases**	**3,110**	**1.87**

*per 1,000 livebirths

**Table 2:** Etiology of recognized syndromes and associations in the entire HCAR datatset and among the subjects with OCs.

**Etiological Entity**	**Entire Dataset**	**Subjects with OCs**
*Mendelian Syndromes*		
Stickler type I*	4	2
Faciogenitopopliteal	2	2
Ectrodactyly, ectodermal dysplasia, CL/P	6	6
Diastrophic displasia	2	1
Meckel	28	4
Orofaciodigital type II	5	5
Roberts	4	4
Orofaciodigital type I	9	1

**Subtotal**	**60**	**25**

*Chromosomal*		
Trisomy 13	35	29
Trisomy 18	22	1
Deletions	25	1

**Subtotal**	**82**	**31**

*Teratogenic*		
Hydantoin	4	4

*Associations*		
Schisis	130	73

**Total**	**276**	**133**

*Stickler syndrome included Robin sequence

Most cases with unidentified syndromic orofacial clefts had a total of 2 malformations. There were 181 subjects with unidentified syndromic CL/P and only 1 other malformation (or 51.6% of all unidentified syndromic CL/P). Similarly, there were 81 subjects with unidentified syndromic CP and only 1 other malformation (or 47.9% of all unidentified syndromic CP) (Table 3). In unidentified syndromic CL/P cases, the most frequent combinations were with anomalies of the heart or circulatory system (*n *= 37, rate: 7.2/100,000 births), club foot (n = 21, rate: 3.1/100,000 births), congenital hydrocephaly (*n *= 22, rate: 3.0/100,000 births), and polydactyly in hand or foot (*n *= 18, rate: 3.0/100,000 births). The most common combinations in those with unidentified syndromic CP were anomalies of the heart or circulatory system (*n *= 24, rate: 4.5/100,000 births), club foot (n = 16, rate: 2.4/100,000 births), congenital anomalies of the ear (*n *= 4, rate: 1.4/100,000 births), and anomalies of the skeletal system, especially spine, ribs, and sternum (*n *= 3, rate: 1.1/100,000 births).

**Table 3:** Frequency (and percentage) of anomalies in cases with non-isolated CL/P and CP of unidentified etiology.

**Total Number of Anomalies**	**Unidentified Etiology CL/P**		**Unidentified Etiology CP**	
2	181	51.6	81	47.9
3	81	23.1	31	18.3
4	40	11.4	28	16.6
5	21	6.0	17	10.1
6	16	4.5	8	4.7
7 or more	12	3.4	4	2.4

**Total**	**351**	**100.0**	**169**	**100.0**

There were 81 and 31 patients with a total of three congenital anomalies including syndromic unidentified CL/P and CP, respectively (Table 3). In these cases, the most common coexisting anomalies for CL/P were of the heart and circulatory system (*n *= 17), polydactyly (*n *= 13) and reduction of the limbs (*n *= 12), while only anomalies of the heart and circulatory system were frequent among those patients with CP (*n *= 11). One hundred and seventy one subjects with unidentified CL/P and 88 with CP had four or more anomalies; however, there were few repeated combinations:

2 cases with CL, congenital heart defect, polydactyly, and hydrocephaly,

2 cases with CP, polydactyly, anorectal atresia/stenosis, and branchial anomalies,

2 cases with CP, hydrocephaly, and anomalies of the skeletal and digestive systems, and

2 cases with CP, anomalies of the diaphragm and of the ear and renal agenesis/dysgenesis.

Table 4 presents the frequency of malformations by affected organ system in children with multiple congenital anomalies and orofacial clefts. Malformations of the skeletal system were the most common in both CL/P and CP subjects, followed by CNS and cardiovascular among the former and cardiovascular and CNS and urogenital in the latter.

**Table 4:** Frequency (and percentage of the total) of malformations by affected organ systems in subjects with multiple congenital anomalies.

	**CL/P (*n *= 436)**	**CP (*n *= 217)**	**Both (*n *= 653)**
Central nervous system	158 (20.6)	44 (11.1)	202 (17.4)
Eye	36 (4.7)	7 (1.8)	44 (3.8)
Ear	32 (4.2)	23 (5.8)	55 (4.7)
Face-neck	16 (2.1)	22 (5.5)	38 (3.3)
Cardiovascular system	119 (15.6)	75 (18.9)	194 (16.7)
Respiratory system	4 (0.5)	4 (1.0)	8 (0.7)
Digestive system	51 (6.7)	30 (7.5)	81 (7.0)
Urogenital system	88 (11.5)	44 (11.1)	132 (11.3)
Skeletal (including limb deficiency)	191 (25.0)	121 (30.5)	312 (26.8)
Skin	0 (0.0)	1 (0.2)	1 (0.1)
Abdominal wall/diaphragma	46 (6.0)	15 (3.8)	61 (5.2)
Other^†^	24 (3.1)	11 (2.8)	35 (3.0)
**Total number of malformations**	**765**	**397**	**1,162**

^†^includes congenital inguinal hernia.

## Discussion

The main objective of this article was to present prevalence and baseline characteristics of cases with syndromic OCs and associated anomalies in the HCAR dataset. The HCAR is an excellent source of cases because it is (1) population-based, (2) from an ethnically homogeneous, well-defined population, (3) with a high recorded birth prevalence of cases with congenital anomalies compared to other registries (approximately 4%) indicating a nearly complete ascertainment, and (4) the clinical diagnoses have a high level of precision [12]. However, the HCAR has two major weaknesses: First, the information on the cases is based on the notification made by several thousand medical doctors who have uneven experience with children with dysmorphic features. Second, the ascertainment covered a period when many of the craniofacial syndromes known today had not been yet delineated.

Of 653 cases with OCs and multiple congenital anomalies, only 133 (20.4%) were part of a known etiological entity (Table 2). Among the 60 Mendelian syndromic cases in the entire HCAR data set, 25 included OCs (41.7%). During the ascertainment period (1973–1982), over 100 syndromes including OCs had been reported in the literature [13]. However, many were not diagnosed and/or notified to the HCAR. Two examples are the Van der Woude syndrome, which is a relatively common autosomal dominant condition with CL/P [14] and is absent in the HCAR and the oculo-auriculo-vertebral spectrum (previously known as Goldenhar syndrome), frequently associated with orofacial clefts, also non-existent in the HCAR. In other cases, there seems to be an over-reporting of certain conditions. For example, there are 28 cases of Meckel syndrome in the HCAR, with 4 cases including OCs, a rare combination.

The proportion of reported chromosomal abnormalities to the HCAR is lower than expected and orofacial clefts are no exception. This might be due to any of the following three facts: (1) although chromosome analysis was recommended in all cases with multiple congenital anomalies, this advice was rarely followed, (2) cases with multiple congenital anomalies had a high perinatal mortality and karyotyping was seldom performed in these cases, and (3) recently developed sensitive chromosomal diagnostic techniques, such as fluorescent in situ hybridization (FISH), were not available during the study period. Therefore, the relatively common deletion 22q11.2 seen in patients with OCs (previously known as velocardiofacial syndrome or Di George syndrome) could not be identified. This might explain our finding of chromosomal anomalies in only 4.7% of the cases with MCAs, significantly less than other population-based studies (ie, Tolarova and Cervenka: 8.8% [7], Stoll et al.: 7.8% [15]).

All four cases caused by teratogens were identified as fetal hydantoin syndrome (Table 2). Fetal alcohol syndrome was notified very rarely to the HCAR. Among cases with diabetic embryopathy, orofacial clefts were not recorded and therefore are not included in this report. The collection and analysis of information on pregnancy history, including maternal drug use, would help identify known or new syndromes caused by teratogens. This was the main motivation for the establishment of the Case-Control Surveillance Program of Congenital Abnormalities in Hungary [9].

Of 130 cases with the schisis association, 55 (42.3%) had CL/P and 16 had CP (12.3%). This association had been originally described by one of us when it was noted that there were cases with neural tube defects (anencephaly, encephalocele, spina bifida cystica), OCs, omphalocele, and diaphragmatic hernia associated with one another far more frequently than at the expected random combination rates [16]. No other association with OCs was found.

The evaluation of these results suggests that the Hungarian registry probably under-reported chromosomal abnormalities. This might be due to the following: first, the HCAR is a cross-sectional registry; thus, its is limited in its ability to obtain detailed clinical descriptions of each infant with a syndrome, including X-rays, karyotypes, or screening for the 22q11.2 deletion. Second, HCAR ascertained cases between 1973 and 1982. The development of improved molecular and cytogenetic tools in the 1980s, which led to the identification of the etiology of many conditions of previously unidentified origin, might account for some misclassification.

## Conclusion

The description of component anomalies in cases with multiple congenital anomalies may help identify recognizable entities and delineate new syndromes. This knowledge can be used to better understand the needs of the population (ie, diagnosis, prognosis, counseling) and to develop policies for health care. In order to be successful, birth defects surveillance systems must include experienced dysmorphologists up-to-date with the latest diagnostic tools and definitions [1]. Engagement with the large multinational registries, such as the International Collaborative Research on Craniofacial Anomalies Project supported by the World Health Organization (WHO) [17], would be of benefit to all as well. It behooves the readers to note that in the HCAR dataset, which contains close to 66,000 congenital anomalies (4% of the total live births), almost 10% of these had more than one anomaly and more than half of these could not be allocated to a particular syndrome or association. This in itself points to the need for a global effort to improve the sensitivity and specificity of diagnosis. It is our hope that the information displayed in this paper will contribute to increase that awareness.

## Competing interests

The author(s) declare that they have no competing interests.

## Authors' contributions

AS, DFW, and AEC conceived the study and participated in its design and coordination and drafted the manuscript. AEC coordinated the overall collection of data. All authors read and approved the final manuscript.

## Pre-publication history

The pre-publication history for this paper can be accessed here:


